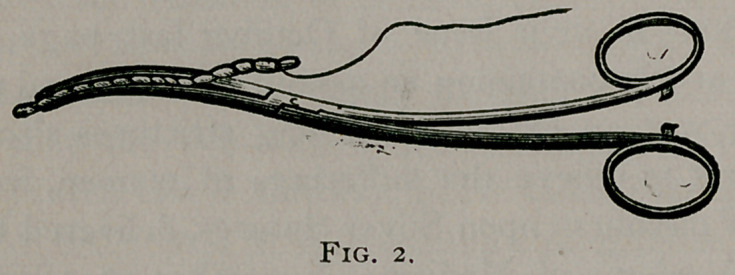# Taliaferro and Bozeman Controversy

**Published:** 1887-03

**Authors:** Nathan Bozeman

**Affiliations:** 307 Fifth Avenue, New York


### TALIAFERRO AND BOZEMAN CONTROVERSY.

BOZEMAN'S UTERINE FORCEPS—DR. KOLLOCK'S, MR. BAKER BROWN'S AND PROFESSOR SIMPSON'S ESTIMATES OF THE EARLIER OPERATIONS FOR VESICO-VAGINAL FISTULE—REMARKS.*

*To the Editors of the Atlanta Medical and Surgical Journal:*

GENTLEMEN—In your issue of October last, page 465, there appeared an article containing an assault upon me and my labors. In this article was an extract containing strictures also upon my humble efforts to relieve the sufferings of women, from Dr. J. Marion Sims' discourse upon Silver Sutures, delivered before the New York Academy of Medicine, November 17, 1857. Knowing well the object and the devious methods of the writer of this article, I was less surprised at the character of the attack upon me than I was at the publication of such an article in a reputable journal.

This man for several years past has evidently felt the necessity of keeping himself before the public, being the head of a private infirmary. His favorite manner of doing this has been by lavishing fulsome praises upon Dr. Sims and by showering opprobrious epithets upon my head, each time appropriating one or more of my instruments and special methods of treatment. As proof of his recent exploit in this latter particular, and of his draft upon my surgical resources without acknowledgment, I will mention his theft of my uterine forceps, an instrument well known to every respectable instrument-maker in New York city and, I venture to say, to the profession generally throughout the country.

FIGURE 1. Bozeman's (uterine) Dressing Forceps, the cut No.

*The number of the journal containing the article here noticed did not reach the writer until the latter part of December. This and sickness since are the causes of the lateness of his reply.

285, the same used by Messrs. Geo. Tiemann & Co., for illustration in their catalogue, part iii., page 74, for the past twelve or fifteen years.

FIG. 1.

FIG. 2.

FIG. 2. Shows an exact copy of the same instrument, in the article under consideration, cunningly purloined by the author under the designation of "Dressing Forceps (with small blades)," with the difference *only* of holding in its grasp "a little pledget or roll of cotton with a small thread wound about it," ready for use.

This omission of "Bozeman's," in the descriptive paragraph referred to, before "dressing forceps," disproves beyond question ignorance of my admitted and published right to the instrument in question, and the explanation, "with small blades" in parenthesis, proves positively, first, helplessness in the execution of the procedure indicated without the aid of my forceps, and, secondly, a fixed determination to steal the credit of the instrument rather than to honorably acknowledge indebtedness for the use of the same—an act in a physician, or an author claiming to be a physician, equaled only by that of his ordering the manufacture of a certain instrument, or instruments, and then of permitting the bill for the same to be outlawed.

To a note I addressed to Messrs. F. G. Otto & Sons, No. 345 Fourth Avenue, New York (formerly Messrs. Otto & Reynders), with regard to the time they first manufactured my forceps, they responded December 30, 1886, as follows:

"In reply to your favor of the 28th instant, our Mr. F. G. Otto recollects making your forceps, known as 'Bozeman's Uterine Forceps,' for about fifteen years past, and it may date back to the year 1870."

The three old nurses of the Woman's Hospital, who have for years had charge of all the hospital instruments, on being shown THE JOURNAL cut of Fig. 2, recognized it at once as being illustrative of my forceps in use there.

It is not my purpose to refer here to the strictures upon me contained in the extract from Dr. Sims' discourse on "Silver Sutures," a refutation of these being already in preparation for publication. Nevertheless, this subject having been revived again after nearly thirty years, and as there are doubtless many of your readers who would like to know something of the real differences that brought Dr. Sims and myself into collision at that early day with regard to the treatment of vesico-vaginal fistulæs—a period when suffering women filled the hospitals of the large cities, appealing to the sympathies of helpless surgeons, and when the cure of even a simple case, by whatsoever method, was regarded as a great triumph—I will simply content myself with appending the concluding remarks of a paper on "The History of Vesico-Vaginal Fistula," read before the State Medical Society of Georgia, at its annual meeting in Augusta, on April 8, 1857, by the late Dr. P. M. Kollock, of Savannah, whose name every physician and gynæcologist of Georgia may well feel proud to honor:

"I have thus detailed three cases occurring in my own practice and treated by suture on the two principles which may now be regarded as most worthy of confidence, and I think it will be conceded that I may, without very great presumption, claim the right to testify in regard to their respective merits. It is to be remarked that in the treatment of these three cases, nine operations by suture were performed, seven by the clamp suture of Dr. Sims and the other two by the button suture of Dr. Bozeman.

"The clamp suture failed in every instance to effect a cure, even in the two cases which seemed as favorable for its success as could be desired. The button suture succeeded perfectly in both cases on the first trial. The preference must, therefore, be given without hesitation to the latter, and I fully endorse the statement of its discoverer, who claims for it the following advantages:

"1. It protects the edges of the fistulous opening against the irritation of the urine, of the vaginal discharges and the atmosphere.

"2. It prevents the wires from cutting out.

"3. It acts the part of a splint in keeping the approximated edges in close contact and at rest.

"I consider this suture the greatest improvement that has ever been made in the treatment of this class of cases. The surgeon can now approach them with a confidence of success before unknown. The profession and the public owe to Dr. Bozeman a debt of unspeakable gratitude. He has achieved an exploit of which he has more reason to be proud than if he were the hero of an Austerlitz or a Waterloo."

Mr. Isaac Baker Brown, of London, after trying for years all other known European methods of operation for vesico-vaginal fistule with only partial and insignificant results, adopted my method and cured a case at the first trial (*Lancet*, p. 540, Nov. 15, 1856). In a paper read by Mr. Brown before the British Medical Association at its annual meeting at Edinburgh, July 31, 1858, which I attended, Mr. Brown said:

"A few months since I published three more cases, two of which were cured by Dr. Bozeman's plan and one by that of Dr. Hayward, of Boston (*Medical Times and Gazette*, Vol. xxxvii, p. 398, April 17, 1858), and now within a few weeks I have succeeded in curing seven more, and all by Dr. Bozeman's method." Mr. Brown finished his report as follows:

"These cases call for little remark. They prove what I stated in the beginning of the pamphlet, that this intractable lesion is now quite under our control. I am so satisfied of this that I am now operating upon cases which formerly I rejected as being incurable, and for whom I feared no operation would be of any avail. . . . . . . . . . . .

"Before concluding this pamphlet I would wish to make a few remarks upon the origination of the metallic wire sutures. The evening previous to my reading these cases before the British Medical Association at Edinburgh, my friend, Professor Simpson, informed me that Mr. Gossett, of the city of London, had published in the *Lancet* for 1834 the history of a case of vesico-vaginal fistula which he had cured by using golden (silver gilt) sutures, and that he also recommended their use in many other surgical cases. The merit of being the first to apply metallic sutures to these cases is therefore undoubtedly due to Mr. Gossett, of London, and not to Professor Sims, of New York. I must further add, however, that I attribute the rapid success of these operations to the use of the button, as first suggested by Dr. Bozeman, and that to him, therefore, is fairly due the merit of rendering this most troublesome lesion comparatively easy of cure."

Mr. Brown honored me with the dedication of the reprint from the transactions of the society in these words:

"MY DEAR DR. BOZEMAN—I dedicate this little pamphlet to you to show how highly I appreciate your great surgical skill in having brought the operation for vesico-vaginal fistula to the highest perfection, and also as a slight proof how much I esteem you personally as an earnest worker in the path of true scientific surgery, as well as a warm, earnest, true-hearted friend.—*17 Connaught Square, Nyde Park, London, August, 1858.*"

On August 15, sixteen days after Mr. Brown read the foregoing paper, while I was still in Edinburgh, Professor James Y. Simpson, then regarded as the highest authority in Europe on diseases of women, invited me to operate upon one of his private patients suffering from a vesico-vaginal fistula, Mrs. W., from Loch Lomond, aged 34. Professor Simpson frankly told me that until hearing Mr. Brown read his paper, he had disbelieved the various published statements of the results achieved by my operation, as they seemed to him to be exaggerated. He had since changed his views regarding them, and wished to learn the details of the operation from me. I performed the operation for him in the presence of several other physicians, Drs. Keiller, Alex. Simpson, Coghill and Mr. Edwards, of Edinburgh, and Dr. Paul, of Elgin. The suture apparatus was removed on the ninth day, and the result was a complete cure. This was the first case of vesico-vaginal fistula, as I learned, ever cured in Edinburgh by operation, except by cauterization.

For an account of other operations performed by me in Europe during this visit, see the *Edinburgh Medical Journal*, the *Glasgow Medical Journal* for October, 1858, and the *Gazette des Hopitaux*, Paris, for January 4 and 6, 1859.

That same form of suture so successfully used by Dr. Kollock and Mr. Baker Brown, and first described by me (*Louisville Review*, May, 1856), together with its associated auxiliary of gradual preparatory treatment for cicatricial complications (incisions and graduated vaginal and uterine pressure). Embodying as it did some of the most important principles of Listerism, as taught in the schools of to-day, formed a notable era in the history of vesico-vaginal fistula. The method then given to the profession, supported as it was in its claims to originality by seven consecutive and complete vesico-vaginal fistulous closures, from May 10 to October 18, 1855, was an unprecedented achievement attained by no other surgeon in the world. The same method from that period to the present time, without change of form or principle, nearly 32 years, has given me an average of one complete fistulous closure at one operation and a half, with diversion of the menstrual flow from its normal outlet in only two cases. Well supported, then, is the statement of Dr. Emmet, one of the highest living authorities upon the subject, in his able work on the "Principles and Practice of Genæcology," third edition, p. 819, 1884, which is to this effect:

"Dr. Bozeman has long since reached a degree of dexterity and skill in this operation (vesico-vaginal and recto-vaginal fistula) by which nearly every case is cured, if the condition of the tissues will admit of this being done without sacrificing the generative functions."

The quotations given from the distinguished surgeons I have named are sufficient refutation of the malicious charges that in this instance have been made against me. I should not have deigned to notice them had they not appeared in so widely read a publication as THE ATLANTA MEDICAL AND SURGICAL JOURNAL. The attack appears to be inspired by sheer envy on the part of its author, who shows himself entirely unmindful of the ethics of the profession in his wanton and indefensible course. He has thus put himself outside the pale of decency. He seeks to build up a reputation by associating his unknown name with those who have achieved eminence by their genius and their originality in their methods of practice. It is annoying to be brought in contact or conflict with persons of this variety, but sometimes there is no escape from them.

The gross impugnment of my honor and the detraction from my rights which have been made in your columns justify me in the hope that you will publish this rectification.

Yours respectfully, NATHAN BOZEMAN.

## Figures and Tables

**Fig. I. f1:**
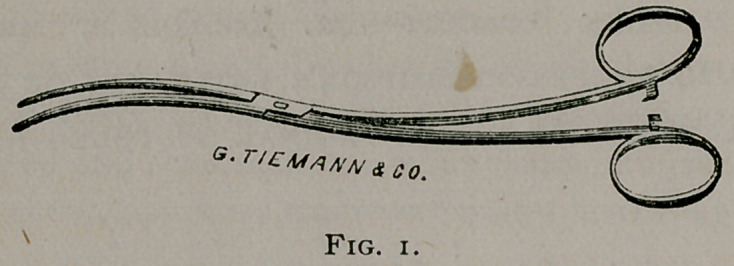


**Fig. 2. f2:**